# The Algorithms of Distributed Learning and Distributed Estimation about Intelligent Wireless Sensor Network

**DOI:** 10.3390/s20051302

**Published:** 2020-02-27

**Authors:** Fuxiao Tan

**Affiliations:** College of Information Engineering, Shanghai Maritime University, 1550 Haigang Ave, Shanghai 201306, China; fuxiaotan@gmail.com; Tel.: +86-21-3828-2800

**Keywords:** intelligent wireless sensor network, distributed topological structure, distributed estimation strategy, distributed learning

## Abstract

The intelligent wireless sensor network is a distributed network system with high “network awareness”. Each intelligent node (agent) is connected by the topology within the neighborhood which not only can perceive the surrounding environment, but can adjusts its own behavior according to its local perception information to constructs a distributed learning algorithms. Therefore, three basic intelligent network topologies of centralized, non-cooperative, and cooperative are intensively investigated in this paper. The main contributions of the paper include two aspects. First, based on algebraic graph, three basic theoretical frameworks for distributed learning and distributed parameter estimation of cooperative strategy are surveyed: increment strategy, consensus strategy, and diffusion strategy. Second, based on classical adaptive learning algorithm and online updating law, the implementation process of distributed estimation algorithm and the latest research progress of above three distributed strategies are investigated.

## 1. Introduction

With the development of automation and wireless communication technology, data acquisition, data processing, data transmission, data control and data storage are not only convenient and fast, but also safe and reliable in a complicated network [1,2]. Combining distributed computing, computer science, automatic control theory, wireless sensor, and microelectronics manufacturing, intelligent network is a kind of large-scale distributed network systems, which is the integration of data-aware, intelligent learning, dynamic optimization, and wireless data communication [3,4]. There are many kinds of intelligent networks, which mainly includes wireless sensor network, cognitive network sensing, cognitive radio network, cognitive radar, smart grid, multi-robot network and so on [5]. Intelligent networks are an emerging interdiscipline, which not only involves machine learning, artificial intelligence, sensor networks, cognitive radio, pattern recognition and optimization theory, but also has a very high application prospect and research value [6,7].

In intelligent wireless sensor network (IWSN), intelligent nodes (agents) are connected through the neighborhood within the network topology. Therefore, the real-time online information processing, data analysis, and dynamic optimization of agents are accomplished [8,9]. Intelligent network is a highly intelligent distributed network system, in which each agent is able to self-recognition, self-judgment, and self-adjusting by network topology [10]. At individual level, intelligent network requires each agent not only can perceive its environment information, but also can perform network communication through its connection to other agents to realize ongoing reinforcement learning [11]. In the organization of the system characteristics, agent can adjust their behavior according to its local “awareness” information [12].

Intelligent nodes are terms such as autonomous agents, which mainly include sensors, processors, actuators, and etc. With the aid of algebraic graph theory, theses nodes are not only communication by network topology, but also allow us to exchange information within their neighborhood and to accomplish network global tasks by online learning [13]. So the IWSN node with distributed manner have “network awareness”, which constructed complicated intelligent network systems. These “network awareness” have the main four characteristic [3]: (1) data awareness, (2) spatial awareness, (3) group awareness, and (4) context-awareness. Therefore, combined machine learning with computational intelligence algorithm, the ISWN integrate with the technology of parameter estimation, adaptive filter, machine learning, online sparse kernel algorithm, system identification, cooperative control of multi-agent systems, distributed optimization, differential game, deep reinforcement learning, and intelligent data analysis.

IWSN has character of scalability and expandability [14]. Machine learning and artificial intelligence algorithms have become the new frontier toward analyzing intelligent network [15]. Therefore, the revolutionary tools of big data processing is critical to expanding the territory of this intelligence system paradigm [16]. For communication nodes and communications links, intelligent network has also robustness [17].

There is a large amount of data in IWSN, which have the following characteristics: large volume of data, various data types, and low-value density of data. Furthermore, the collecting, analysis, saving, and fast processing real-time data can be conducted by agents with intelligent sensors in the intelligent network [18]. Under a big data environment, agents are constantly sharing and there is diffusion of information through the network topology among intelligence nodes, which can not only monitor uncertain real-time data, but also can dynamically adjust the network topology. Therefore, in large volume and various data, it is an important research direction of distributed estimation and distributed learning, in which machine learning can efficiently excavate the laws hidden in the big data to design a distributed dynamic optimization algorithm [19].

Since the extensive application of various algorithms in IWSN, the amount of data and the complexity of data are increased correspondingly [20]. Modern computing equipment is a very complex system, which enables it to store more complex data and to deal with a larger amount of data. Then, the computational complexity is also increasing simultaneously [21]. In addition, a variety of running programs in IWSN can process many complex data streams to study prediction models and can extract the inherent model from noise data. In order to ensure data resolution and to avoid data loss, it is necessary to analyze the data flow in IWSN in real time [22]. Therefore, massive, multidimensional, and highly speeding data streams make it impractical to use existing learning and filtering algorithms to analyze real-time data [23].

In terms of big data analysis, behavioral pattern recognition, and information evolution, the reason for the rapid development of machine learning is its ability to describe potential relationships among large-scale data which can effectively solve all kinds of complex problems [24,25,26]. Furthermore, in the process of machine learning, based on the potential mapping relationship between expected output data and input data, the commonly used machine learning algorithms can be divided into three types: unsupervised learning, supervised learning, and reinforcement learning [27]. So, with the advent of the era of cloud computing and big data, the training inefficiency can be alleviated by the large increase of computing ability and the risk of over-fitting can be reduced by the large increase of training data [28]. Therefore, as the representative of large-scale learning algorithms such as broad learning [29] and deep learning [30] have been getting more and more attention. Moreover, as a type of the reinforcement learning algorithm, adaptive dynamic programming has achieved gratifying results in the design of optimal control system [31,32,33] and other control-related issues [34,35,36,37,38].

IWSN has highly cognitive function [39,40]. Using distributed collaboration of agents, each intelligent node is able to communicate with other intelligent nodes in the neighborhood which have access to environmental information and to process and to manage a large amount of real-time data in the intelligent network [41,42]. Thus, the relevant distributed estimation algorithm can be designed and the sparse processing and dynamic optimization of IWSN can be realized [43]. Furthermore, the quickness, real-time, accuracy, and reliability of data transmission can be ensured simultaneously in the realization of network connectivity [44,45].

In conclusion, by integrating with signal processing, wireless sensor network, machine learning, data-sparse algorithm, dynamic optimization, and control theory, investigating and developing distributed learning and distributed estimation over networks has become an important problem in the practical engineering application [46,47]. Furthermore, in the true environment of IWSN, distributed estimation algorithm could be able to deal with some problems of uncertainty phenomenon, which mainly includes the dynamic change of topological structure, quantization errors, communication link failure, packet losses, serious distortion of communication channel, and inter-symbol interference (ISI) [48,49].

So, it is very urgent to analyze the latest research progress on cooperation, noncooperation, real-time adaptation, online learning, self-healing, and self-organization about intelligent networks. On this basis, the completed results of scientific research in recent years have been surveyed and the future research direction has also been prospected.

The theoretical framework of “Distributed Learning and Distributed Estimation” includes the following point. (1) Three basic theoretical frameworks for distributed learning and parameter estimation of intelligent network topologies are intensively investigated: centralized topology, non-cooperative topology, and cooperative topology. (2) Based on algebraic graph, three basic cooperative strategies are proposed: increment strategy, consensus strategy, and diffusion strategy. (3) Based on the classical adaptive learning algorithm and online updating equation, the algorithm implementation process and the latest research progress of the above three distributed strategies are intensively studied.

The structure of the paper is as follows. Section 1 is introduces the intelligent network. In Section 2, the main distributed topology of intelligence network is discussed. Section 3 introduces three distributed strategies and the online updating rule. Section 4 describes the implementation of a distributed learning algorithm in detail. Finally, the conclusion and future research perspective are in the Section 5.

## 2. Basic Topologies of IWSN

Because of power supply, computing complexity, communication bandwidth, and limited resources, the IWSN has been restricted in practical applications and this has affected the future development [50]. Thus, in order to solve these problems, IWSN should be designed according to the following principles [51].

(1) Development of intelligent sensor nodes with awareness that can conduct self-identification and self-judgment of each agent.

(2) Development of distributed adaptive dynamic optimization algorithm that can be able to online learning and distributed estimation of intelligent network.

(3) Development of Ad Hoc network with awareness that the connectivity of intelligent networks can be guaranteed under dynamic topology.

The IWSN inevitably has various dynamic problems in practical application. Under the condition of limited resources and limited time, IWSN needs to solve a series of problems with complex real-time dynamic environment of coordination, conflict resolution, network resource allocation, and task scheduling [52]. So, by building intelligent network system based on intelligent sensors, each agent can update their environmental information constantly. However, the disturbances of environmental information can cause the dynamic change of multi-agent’s behavior and even lead to instability [53]. Thus, under the influence of various factors, the intelligent network will produce structure changes and cause the dislocation of network or information loss [54].

**Figure 1:**
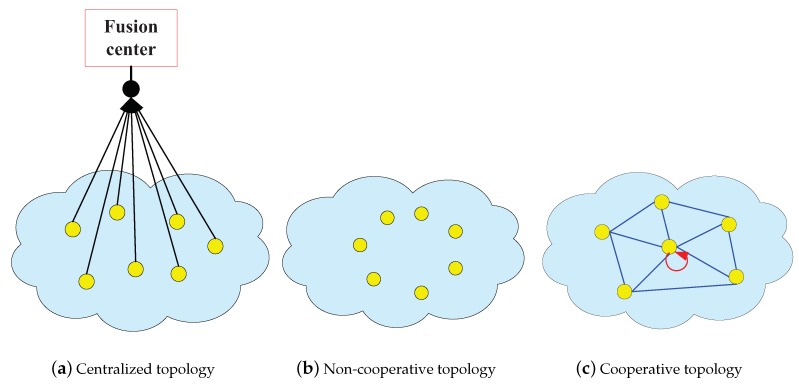
The topology of intelligent wireless sensor network (IWSN).

Based on the topology of communication, there are three basic structures of IWSN: centralized topology, non-cooperative topology, and cooperative topology, which are shown in the Figure 1. Figure 1a is centralized topology. Figure 1b,c are non-cooperative topology and cooperative topology respectively.

### 2.1. Centralized Topology of IWSN

In the centralized topology, intelligent network has a data fusion center. Thus, each intelligent node can send data to the fusion center respectively. Furthermore, the characteristic of data fusion is centralized data processing, in which the collection and processing of various intelligent transmitted data can be achieved [55]. Then, the fusion center performs computations and sends the processed data back to each agent. The centralized topology is shown in Figure 1a.

While the centralized structure has a powerful information processing center and effective transmission of data over the topology, the centralized topology has its limitations [56]. First of all, in the real-time communication system, agents collect a large amount of data continually and exchange data between the data fusion center and each agent. Because these communications are all wireless communication mode, it needs some important dynamic source routing. So, the manufacturing cost is very expensive. Secondly, because of the needs of privacy and secrecy, each agent will not share all of its own data to the data fusion center in the highly intelligent wireless sensor system. What’s more important is the centralized topology has a critical flaw. When the data fusion center is faultly, all the data will not be able to transmit timely and effectively which will give rise to the whole network system breakdown directly.

### 2.2. Cooperation Topology and Non-Cooperative Topology

In practical engineering, the IWSNk is generally designed by the distributed topology structure. For the distributed topology, each agent is linked with each other through a certain topological structure, which ensure to achieve information sharing and to transport information effectively among agents and their neighborhood agents. In general, the distributed topologies can be divided into two categories: cooperation topology and non-cooperative topology [50].

In non-cooperative topology, agents are all independent of each other to pursue their own expectations. Each agent is sharing data and its behavior by itself [57,58].

However, today’s all kinds of existing distributed network, such as internet network, smart grid, traffic network, wireless sensor network (WSN), biological information network mostly exists in the way of cooperation [59]. For the real network system, on the one hand, intelligent network adopt cooperation strategy to improve the system of optimality, to enhance network robustness, and to strengthen the fault self-recovery ability. Therefore, the cooperative strategy is more humanization and personalization in privacy and confidentiality. On the other hand, in the decentralized location, each agent can be easier to get a lot of online data, which can increase the distributed information processing capacity of the network. Furthermore, using the distributed topology, agent can process data for data analysis and data mining, which not only improve the learning ability of the network, but also provide a very effective method for distributed estimation of IWSN [60].

**Figure 2:**
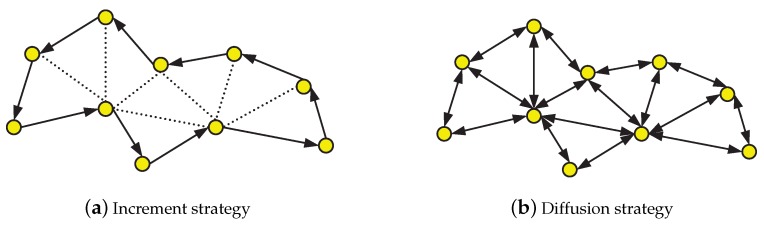
The IWSN with the distributed cooperative strategy.

The IWSN with distributed cooperative strategy is shown in Figure 2, where the Figure 2a is increment strategy and the Figure 2b is the diffusion strategy.

## 3. Cooperative Distributed Estimation Strategy

Algebraic graph theory is an important branch of graph theory, which mainly uses algebraic methods and results to study related scientific problems by graph theory [61]. Therefore, algebraic graph theory is the theoretical basis for the analysis of IWSN. This means that each intelligent node (agent) is the vertex of the graph and the edge in the graph represents the communication structure between agents [62]. In order to study the topological construction and topological properties about intelligent network, some matrix theories about graph theory are introduced, which main include the adjacent matrix of a graph, correlation matrix, and the Laplace matrix, etc. [63]. Furthermore, in the theory of algebraic graph, one of the main research areas is whether and how the topological properties of graphs can be reflected by the algebraic properties of these matrices, so that the algebraic properties of these matrices can be studied by using matrix theory  [64]. Then, the topological properties of IWSN are obtained. Particularly, based on algebraic graph theory, the research on consensus protocol and cooperative control of multi-agent network system is a hot and difficult point in intelligent network system [65].

In IWSN, each intelligent node (Agent) not only can perform data collection and data mining, but also can conduct distributed information processing [64]. Agent collects all kinds of online data related to its own parameters, observation noise, and various data of other agents connected to its topology for online parameter estimation [66]. In this way, if each agent can obtain data of the whole network, the distributed estimation algorithm can accurately estimate various parameters. Obviously, the effective implementation of distributed estimation algorithm is mainly dependent on the cooperation strategies among agents. In the structure of the cooperative strategy, existing literatures are shown that there are three fundamental distributed estimation frameworks: incremental strategies, diffusion strategies, and consensus strategies [50].

### 3.1. The Problem of Distributed Estimation

**Figure 3:**
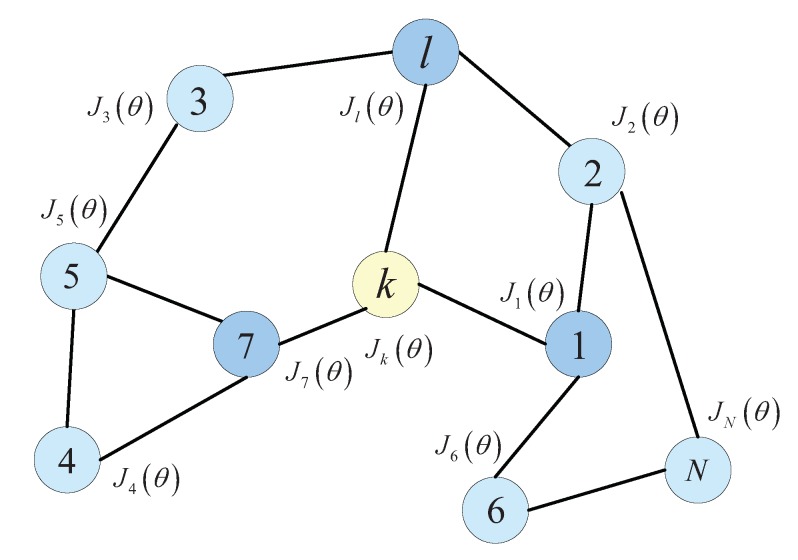
The topology of IWSN with *N* intelligent node.

Consider an IWSN with *N* intelligent node (Agent) over distributed spatial domain, which labeled k=1,2,⋯,N and is shown in Figure 3. The topology of an IWSN is defined as an undirected graph. Thus, let *G* be an undirected graph. The *V* and ε are defined set of nodes and edges respectively. Agent *l* is called a neighbor of agent *k* if agent *k* and agent *l* can receive information from each other, that is $\left(l,k\right)\in \varepsilon$. The neighborhood of agent *k* is denoted by $N_{k}$, which denotes the set of nodes connected to node *k*: $N_{k}=\left\{l\left|\left(l,k\right)\in \varepsilon \right.\right\}$. The agents in the network will estimate an unknown M×1 vector $\theta^{*}$. At each time *i*, each agent *k* can collect a zero-mean scalar measurement $d_{k}\left(i\right)$ and a zero-mean 1×M regression vector $\xi_{k}\left(i\right)$ with a positive-definite convergence matrix $R_{\xi ,k}=E\left[\xi_{k}^{T}\left(i\right)\xi_{k}\left(i\right)\right]>0$, where *E* is the mathematical expectation. Assuming the data $\left\{d_{k}\left(i\right),\xi_{k}\left(i\right)\right\}$ are satisfy the linear regression model [67]

(1) $$d_{k}\left(i\right)=\xi_{k}\left(i\right)\theta^{*}+\varepsilon_{k}\left(i\right),i\geq 0,k=1,\cdots ,N,$$

where $\varepsilon_{k}\left(i\right)$ is a measurement noise with zero-mean and variance $\sigma_{\varepsilon ,k}^{2}=E\left[\varepsilon_{k}\left(i\right)\varepsilon_{k}^{T}\left(i\right)\right]$, which is assumed to be temporally white and spatially independent. The $\theta^{*}$ is optimal estimator.

Assuming the regressors $\left\{\xi_{k}\left(i\right)\right\}$ is temporally white and spatially independent, that is

(2) $$E\left[\xi_{k}^{T}\left(i\right)\xi_{l}\left(j\right)\right]=R_{\xi ,k}\cdot \delta_{kl}\cdot \delta_{ij}$$

in terms of Kronecker delta function

(3) $$\delta_{kl}=\left\{\begin{array}{c} 1,k=l\\ 0,k\neq l \end{array}\right.$$

where the noise $\varepsilon_{k}\left(i\right)$ and the regressors $\left\{\xi_{l}\left(j\right)\right\}$ are assumed to be independent of each other for all $\left\{k,l,i,j\right\}$.

The mean-square-error (MSE) cost function associated with each agent *k* is defined as [68]

(4) $$J_{k}\left(\theta \right)=E{\left|d_{k}\left(i\right)-\xi_{k}\left(i\right)\cdot \theta \right|}^{2}.$$

The main objective of the intelligent network is to estimate $\theta^{*}$ in a distributed topology by the online learning process. Therefore, for estimating $\theta^{*}$, the agents should minimize the following global cost function

(5) $$J^{glob}\left(\theta \right)≜\sum_{k=1}^{N}{E\left|d_{k}\left(i\right)-\xi_{k}\left(i\right)\cdot \theta \right|}^{2}.$$

Supposing the individual agent cost function $J_{k}\left(\theta \right)$ has convex character and the estimation process $d_{k}\left(i\right)$ and $\xi_{k}\left(i\right)$ are jointly stationary, the unique global minimum $\theta_{k}^{*}$ of (5) is well known Wiener filter estimate

(6) $$\theta_{k}^{*}={\left(\sum_{k=1}^{N}R_{\xi ,k}\right)}^{-1}\cdot \left(\sum_{k=1}^{N}r_{d\xi ,k}\right),$$

where $r_{d\xi ,k}≜E\left[d_{k}\left(i\right)\cdot \xi_{k}\left(i\right)\right]$ and $\theta_{k}^{*}$ is optimal estimation of agent *k* [69].

### 3.2. Noncooperative Distributed Estimation Strategy

Based on the traditional stochastic steepest-descent algorithm, agent *k* satisfies the following form to determine the solution [57,58]

(7) $$\theta_{k}\left(i\right)=\theta_{k}\left(i-1\right)-\mu_{k}{\left[\nabla_{\theta }J_{k}\left(\theta_{k}\left(i-1\right)\right)\right]}^{*},$$

where $\mu_{k}>0$ is a constant step size parameter by agent *k* and $\nabla_{\theta }J_{k}\left(\cdot \right)$ is the gradient vector of $J_{k}\left(\theta \right)$ with respect to the variable θ. At time *i*, let $\theta_{k}\left(i\right)$ is the estimate of $\theta_{k}^{*}$ for agent *k*. Under the topology of non-cooperative, each agent attempts to estimate $\theta^{*}$ by itself. Based on any initial condition $\theta_{k}\left(0\right)$, the gradient descent recursive algorithm satisfy the following equation

(8) $$\theta_{k}\left(i\right)=\theta_{k}\left(i-1\right)+\mu_{k}\left[r_{d\xi ,k}-R_{\xi ,k}\cdot \theta_{k}\left(i-1\right)\right],i\geq 0.$$

In order to ensure the convergence of non-cooperative recursive learning algorithm, the $\mu_{k}$ is selected within the interval $\left(0,2/\lambda_{\max}\left(R_{\xi ,k}\right)\;\right)$.

Since the moments $r_{d\xi ,k}$ and $R_{\xi ,k}$ are all stochastic, it is necessary to find a new approach that permit each agent to approximate the unavailable moments $\left\{r_{d\xi ,k},R_{\xi ,k}\right\}$ [70].

In general, one of the simplest methods used is the following instantaneous approximations

(9) $$R_{\xi ,k}\approx \xi_{k}{\left(i\right)}^{T}\xi_{k}\left(i\right),\;\;r_{d\xi ,k}\approx d_{k}\left(i\right)\xi_{k}{\left(i\right)}^{T}.$$

Thus, the corresponding stochastic-gradient recursive algorithm satisfy

(10) $$\begin{array}{cc} \theta_{k}\left(i\right) & =\theta_{k}\left(i-1\right)\\ \; & +\mu_{k}\cdot \xi_{k}{\left(i-1\right)}^{T}\left[d_{k}\left(i-1\right)-\xi_{k}\left(i-1\right)\theta_{k}\left(i-1\right)\right], \end{array}$$

which is the well-known least-mean-squares (LMS) adaptive algorithm.

### 3.3. Cooperative Distributed Strategy

For cooperative strategies, agents are permitted to interact with their neighbors. In this way, the global optimization problem of IWSN can be defined as

(11) $$\begin{array}{cc} \underset{\theta }{minimize}\;J^{glob}\left(\theta \right) & ≜\sum_{k=1}^{N}J_{k}\left(\theta \right)\\ \; & =\sum_{k=1}^{N}E{\left|d_{k}\left(i\right)-\xi_{k}\left(i\right)\theta \right|}^{2}, \end{array}$$

for which $\theta^{*}$ is a unique global optimal solution.

In general, there are three types of cooperative strategies about the intelligent network: increment strategy, consensus strategy, and diffusion strategy [71].

#### 3.3.1. Increment Strategy

**Figure 4:**
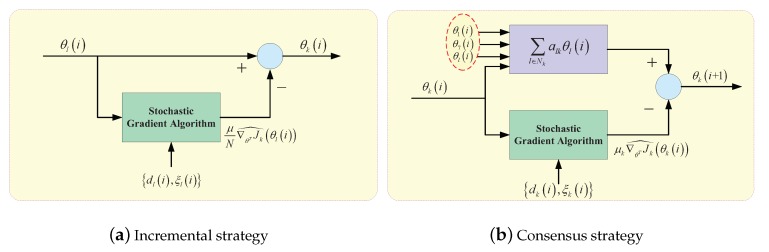
Two types of distributed estimation with cooperative strategy: incremental strategy and consensus strategy. (**a**) A cyclic path with increment strategy is the start of node *l*, in which all intelligent nodes can be visited in Figure 3. (**b**) The neighbors of intelligent node *k* with consensus strategy is defined as $\left\{1,7,l,k\right\}$.

For the incremental strategy, if there is a cycle topology in the intelligent network, the number of agents along the trajectory is from 1 to *N*. In this strategy, the signal is transmitted from one intelligent node to the next node in the cycle edge until all nodes are obtained. The topology of incremental strategy is shown in Figure 4a. Thus, the entirely distributed solution of increment strategy can only access to signal from its local neighbors [72,73].

Therefore, the incremental strategy for online learning is as follows. For each time instant i≥0, the fictitious boundary condition $\theta_{0}\left(i\right)=\theta \left(i-1\right)$ is set. When signal cycle over intelligent nodes k=1,2,⋯,N, intelligent node *k* receives $\theta_{k-1}\left(i\right)$ from its preceding neighbor k-1. At this time, the updating rule of intelligent node *k* satisfy

(12) $$\theta_{k}\left(i\right)=\theta_{k-1}\left(i\right)-\frac{\mu }{N}\hat{\nabla_{\theta^{T}}J_{k}}\left(\theta_{k-1}\left(i\right)\right),$$

where μ>0 is a small step-size and setting $\theta_{i}=\theta_{N}\left(i\right)$ at the end of cycle. According above incremental strategy, the true gradient vector $\nabla_{\theta^{T}}J_{k}\left(\cdot \right)$ is replaced by an instantaneous approximation $\hat{\nabla_{\theta^{T}}J_{k}}\left(\cdot \right)$ [74,75,76,77]. The algorithm implementation of increment strategy for distributed learning is written in Algorithm 1.
**Algorithm 1:** Increment strategy for distributed learning.1:**for** each time i≥0 **do**2:set the fictitious boundary condition at $\theta_{0}\left(i\right)\leftarrow \theta \left(i-1\right)$;3:**cycle** over intelligent node k=1,2,⋯,N;4:intelligent node *k* receives $\theta_{k-1}\left(i\right)$ from its preceding neighbor k-1;5:according to Equation (12), intelligent node *k* online learning;6:**end**7:$\theta \left(i\right)\leftarrow \theta_{N}\left(i\right)$;8:**end for**

#### 3.3.2. Consensus Strategy

In the consensus strategy, each agent *k* performs two steps at each iteration *i*: (1) it aggregates the iteration from its neighbors; (2) updates this aggregate value by negative of conjugate gradient vector evaluated at its existing iterate. The topology of the consensus strategy for the online learning algorithm is shown in Figure 4b.

Given instant time i≥0, each intelligence node k=1,2,⋯,N performs the updating rule of consensus strategy [78]

(13) $$\left\{\begin{array}{c} \psi_{k}\left(i+1\right)=\sum_{l\in N_{k}}a_{lk}\theta_{l}\left(i\right)\\ \theta_{k}\left(i+1\right)=\psi_{k}\left(i\right)-\mu_{k}\hat{\nabla_{\theta^{T}}J_{k}}\left(\theta_{k}\left(i\right)\right). \end{array}\right.$$

For each agent k=1,2,⋯,N, the combination coefficients $\left\{a_{lk}\right\}$ are nonnegative scalars, which satisfy $a_{lk}\geq 0$, $\sum_{l=1}^{N}a_{lk}=1$, and $a_{lk}=0$ if $l\notin N_{k}$ [79].

The above condition means that the combination matrix $A=\left[a_{lk}\right]$ satisfies $A^{T}\cdot 1=1$, where 1 denotes the vector with all entries equal to one. Thus, A is called the left-stochastic matrix [80,81,82]. Therefore, the algorithm implementation process of consensus strategy for distributed learning is shown in Algorithm 2.
**Algorithm 2:** Consensus strategy for distributed learning.1:**for** each time i≥0 **do**2:based on the neighborhood $N_{k}$, intelligent node *k* compute the combination coefficients $\left\{a_{lk}\right\}$;3:according to Equation (13), each intelligent node k=1,2,⋯,N conduct online learning;4:**end for**

It should be noted that the consensus protocol in the networked multi-agent systems is defined by the rules for the interaction of agents in the exchange of information between an agent and its adjacent agent. That is, with the evolution of time, all states of agents in the multi-agent system will tend to be the same point [52,65].

#### 3.3.3. Diffusion Strategy

Generally speaking, there are two basic forms of distributed estimator with diffusion strategy: the adapt-then-combine (ATC) structure and the combine-then-adapt (CTA) structure [50,60,69,71].

**Figure 5:**
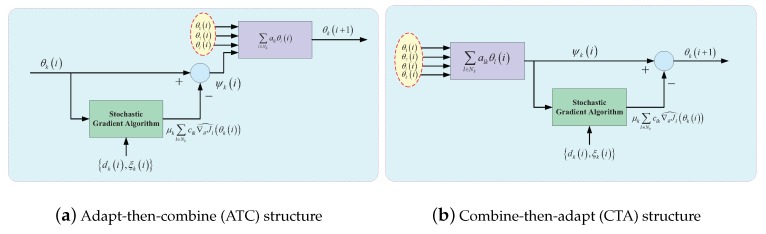
The two structures of diffusion strategy.

The topology of diffusion strategies for online learning algorithm are shown in Figure 5. Figure 5a shows the ATC strategy and Figure 5b shows CTA strategy.

Let $N_{k}$ denote the neighborhood of agent *k*. The optimal estimation with the ATC diffusion strategy needs solve Equation (11). Therefore, for each time instant i≥0, the online learning algorithm of each agent k=1,2,⋯,N with ATC diffusion strategy satisfy

(14) $$\left\{\begin{array}{c} \psi_{k}\left(i+1\right)=\theta_{k}\left(i\right)-\mu_{k}\sum_{l\in N_{k}}c_{lk}\hat{\nabla_{\theta }J_{l}}\left(\theta_{k}\left(i\right)\right)\\ \theta_{k}\left(i+1\right)=\sum_{l\in N_{k}}a_{lk}\psi_{l}\left(i+1\right) \end{array}\right.$$

Furthermore, the online learning algorithm of each agent k=1,2,⋯,N with the CTA diffusion strategy satisfy

(15) $$\left\{\begin{array}{c} \psi_{k}\left(i\right)=\sum_{l\in N_{k}}a_{lk}\theta_{l}\left(i\right)\\ \theta_{k}\left(i+1\right)=\psi_{k}\left(i\right)-\mu_{k}\sum_{l\in N_{k}}c_{lk}\hat{\nabla_{\theta }J_{l}}\left(\theta_{k}\left(i\right)\right), \end{array}\right.$$

**Algorithm 3:** Diffusion strategy for distributed learning (ATC).1:**for** each time i≥0 **do**2:based on the neighborhood $N_{k}$, intelligent node *k* compute the combination coefficients $\left\{a_{lk}\right\}$ and $\left\{c_{lk}\right\}$;3:according to Equation (14), each intelligent node k=1,2,⋯,N conduct online learning;4:**end for**
where $\hat{\nabla_{\theta }J_{l}}\left(\cdot \right)$ is an approximation of the true gradient vector $\nabla_{\theta }J_{l}\left(\cdot \right)$ and $\mu_{k}$ is small constant step-size parameter.
**Algorithm 4:** Diffusion strategy for distributed learning (CTA).1:**for** each time i≥0 **do**2:based on the neighborhood $N_{k}$, intelligent node *k* compute the combination coefficients $\left\{a_{lk}\right\}$ and $\left\{c_{lk}\right\}$;3:according to Equation (15), each intelligent node k=1,2,⋯,N conduct online learning;4:**end for**

Therefore, the algorithms of diffusion strategy for distributed learning are shouwn in Algorithm 3 and Algorithm 4. Algorithm 3 is the implementation of ATC, and Algorithm 4 is the implementation of CTA.

In addition, the $\left\{a_{lk},c_{lk}\right\}$ are non-negative coefficient which satisfy the following conditions

(16) $$\left\{\begin{array}{c} a_{lk}\geq 0,\sum_{l=1}^{N}a_{lk}=1;a_{lk}=0,l\notin N_{k}\\ c_{lk}\geq 0,\sum_{l=1}^{N}c_{lk}=1;c_{lk}=0,l\notin N_{k} \end{array}\right.$$

Furthermore, if the coefficients $\left\{a_{lk},c_{lk}\right\}$ are collected into N×N matrices $C≜\left[c_{lk}\right]$ and $A≜\left[a_{lk}\right]$, we can get a right-stochastic matrix and a left-stochastic matrix, respectively.

### 3.4. The Differences among Three Distributed Estimation Algorithms

Based on the non-cooperative strategy, consensus strategy, and diffusion strategy, a unifying online parameter estimation algorithm can be described the above three strategies [50]. According to three sets of $\left\{a_{0,lk},a_{1,lk},a_{2,lk}\right\}$, the unifying equation can be written as [71]

(17) $$\left\{\begin{array}{c} \phi_{k}\left(i\right)=\sum_{l\in N_{k}}a_{1,lk}\theta_{l}\left(i\right)\\ \psi_{k}\left(i+1\right)=\sum_{l\in N_{k}}a_{0,lk}\phi_{l}\left(i\right)-\mu_{k}\hat{\nabla_{\theta^{*}}J_{k}}\left(\phi_{k}\left(i\right)\right)\\ \theta_{k}\left(i+1\right)=\sum_{l\in N_{k}}a_{2,lk}\psi_{l}\left(i\right) \end{array}\right.$$

where $\left\{\phi_{k}\left(i\right),\psi_{k}\left(i+1\right)\right\}$ is M×1 intermediate variables.

In addition, $A_{0}=\left[a_{0,lk}\right]$, $A_{1}=\left[a_{1,lk}\right]$, and $A_{2}=\left[a_{2,lk}\right]$ are defined as N×N matrices with non-negative entries, respectively, which satisfy the condition of [79] simultaneously. Thus, the $\left\{A_{0},A_{1},A_{2}\right\}$ have the left-stochastic property of the matrix, which satisfies $A_{0}^{T}1=1$, $A_{1}^{T}1=1$, and $A_{2}^{T}1=1$. Furthermore, when $l\notin N_{k}$, any combination weight $\left\{a_{0,lk},a_{1,lk},a_{2,lk}\right\}$ will be equal to zero. Therefore, by defining the product of matrix $P=A_{0}A_{1}A_{2}$, the different distributed strategies can be defined by selecting different matrices $\left\{A_{0},A_{1},A_{2}\right\}$. So, the difference among online updating equations of three distributed strategies can be written as [71]

Non-cooperative: $A_{1}=A_{0}=A_{2}=I_{N}\to P=I_{N}$;

Consensus: $A_{0}=A,A_{1}=I_{N}=A_{2}\to P=A$;

CTA diffusion: $A_{1}=A,A_{2}=I_{N}=A_{0}\to P=A$;

ATC diffusion: $A_{2}=A,A_{1}=I_{N}=A_{0}\to P=A$.

### 3.5. Extension Analysis of Distributed Estimation Algorithm

The above-mentioned distributed learning and estimation algorithm of IWSN are based on the condition of constant step size so that the agent can continuously carry out online adaptive learning and distributed estimation according to the data flow. Furthermore, the extension algorithm associated IWSN is basically developed further by these three strategies. Therefore, several other important aspects of the existing intelligent network distributed algorithm are also need to be further studied.

#### 3.5.1. Distributed Strategy with Sparse and Regularization

In the IWSN, agent can have access to real-time online data through its sensor [83]. The unknown parameters of the system are identified from input–output data to construct a recursive algorithm in order to search the optimization strategy and to design adaptive dynamic optimization algorithm [84]. Because there are a lot of online data in the IWSN, when the number of observation sample points of the intelligent network is increasing, the problem of online data-sparse and regularization should be solved [85,86].

#### 3.5.2. Gossip Strategy

In practical IWSN and especially in mobile Ad Hoc networks, because the network topology is dynamically changing, the agent can select a subset of its neighbors for learning [87]. Thus, each agent can be avoided by exchanging information with other agents in its neighborhood without interruption at every moment [88]. Therefore, distributed algorithms may be designed to determine which and how many subsets within the neighborhood are selected and share data with other agents through the selected link. A simple strategy, which is called the gossip algorithm, is to randomly select a neighborhood at each time [89,90].

#### 3.5.3. Asynchronous Strategy

Because the topology of intelligent network is dynamic, there are a lot of uncertain factors, including the arrival time of the random data, the communication fault of the random link, and the communication delay. Therefore, the distributed learning and distributed estimation of intelligent networks cannot achieve full synchronization, so it is necessary to design distributed estimation algorithms under asynchronous strategy [91,92].

#### 3.5.4. Distributed Strategy with Noise

In an intelligent network, the influence of noise is inevitable in the process of information exchange among agents [93]. To establish a mathematical model with noise link, a distributed estimation algorithm with noise can be designed by adding noise component into the iterative algorithm [94,95].

#### 3.5.5. Distributed Kalman Filter

In an intelligent network, the signal is inevitably affected by external interference and equipment internal noise in the transmission process [96]. In order to obtain useful signals and suppress noise, a distributed filtering algorithm needs to be designed [97]. The distributed Kalman filter is a kind of real-time recursive algorithm, which is based on the statistical characteristics of system noise, and observation noise and the systematic observations are used as the input of the filter. The required estimated value (state or parameter of the system) is taken as the output of the filter, which the input and output of the filter are connected by the algorithm of time updating and observation updating [98]. Thus, the useful signals are estimated online according to the state equation and the observation equation of the system [99].

#### 3.5.6. Distributed Bayesian Learning

For the Bayesian learning problem in wireless sensor networks, references [10,13,19] systematically studied how to solve the Bayesian learning problem by using the variational Bayes method in a distributed environment. For the problem of Bayesian inference and estimation on the network, reference [10] proposed a general framework of distributed variational seeing algorithm for conjugate exponential family models. For the joint sparse signal recovery problem in sensor networks, a distributed variational Bayesian algorithm based on quantized communication and inaccurate ADM is proposed in [19]. This algorithm can, not only save traffic, but also achieves fairly good estimation performance and fast convergence speed.

### 3.6. Example

**Figure 6:**
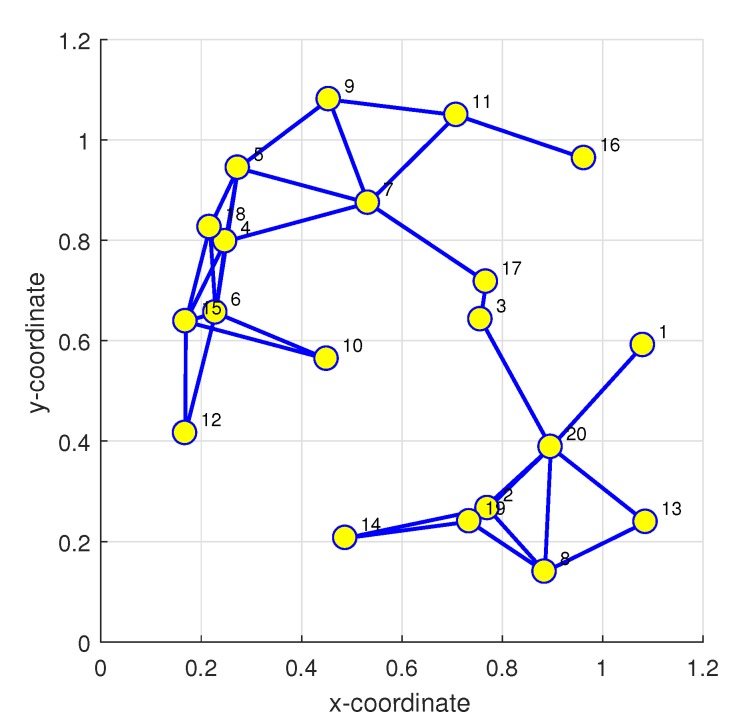
A strongly-connected topology of IWSN with N=20 agents.

A mobile IWSN of strongly-connected topology with N=20 agents is constructed in Figure 6. The ad hoc WSN is generated by the random dynamic network topology with the unity square. Moreover, the mean-square-deviation (MSD) of the stochastic gradient algorithm is defined as the size of the error variance in steady-state mean square value after sufficient iterations [71].

Assuming all agents have uniform step sizes and employ uniform regression covariance matrices $R_{\xi ,k}=R_{\xi }$ for k=1,2,⋯,N and the entries of the target vectors $\theta^{*}$ of size M=10. The noise variance transmits all agents uniformly with white Gaussian noise $\varepsilon_{k}=\sigma_{\varepsilon }^{2}=10^{-2}$. Furthermore, all agents employ the same step-size μ=0.003.

According to the averaging rule [69], the combination weights $\left\{a_{lk}\right\}$ are selected

(18) $$a_{lk}=\left\{\begin{array}{cc} \frac{1}{n_{k}}, & l\in N_{k}\\ 0, & l\notin N_{k}, \end{array}\right.$$

where $n_{k}≜\left|N_{k}\right|$ is the size of the neighborhood about agent *k* (or its degree).

**Figure 7:**
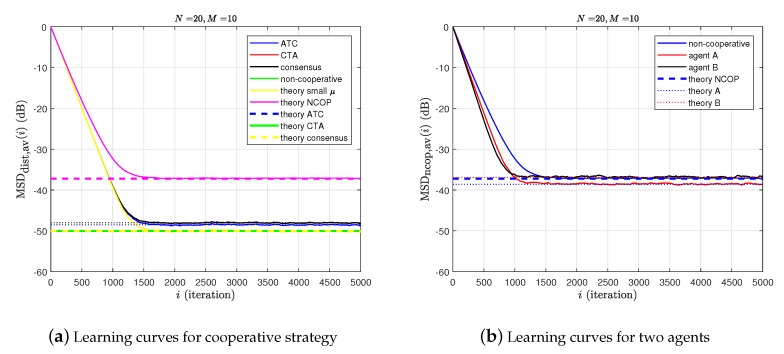
The learning curves for three cooperative strategies: ATC diffusion, CTA diffusion, and consensus.

Based on [71], by executing Algorithm 2, Algorithm 3, and Algorithm 4, the corresponding distributed learning curves for three cooperative strategies: ATC diffusion, CTA diffusion, and consensus are in Figure 7. Figure 7a is learning curves for cooperative strategy and Figure 7b are the learning curves for any two agents.

Furthermore, in the real-time application and experimental tests, the impacts from wireless network channel refer to how to compensate the interference effectively at the receiver, especially the inter-symbol interference (ISI), in order to reduce the bit error rate (BER) of the system, that is, to equalize the distorted channel in the wireless sensor network effectively [100,101]. Therefore, based on the above-distributed strategies and combined with blind algorithm and non-blind algorithm, the channel estimation and equalization theory of wireless sensor networks will be a very interesting research direction [102].

## 4. The Main Results of the Distributed Estimation

In [72], the cooperative mechanism of the adaptive increment strategy is investigated and future research directions are also discussed. Based on the affine projection algorithm, in [73], an adaptive increasing learning algorithm is designed and the algorithm implementation process in the intelligent network is also analyzed. In view of the spatially distributed network, in [74], two kinds of distributed estimation algorithms have been designed: incremental least-mean-square (ILMS) algorithm and spatial least-mean-square (SLMS) algorithm, where the advantages and disadvantages of each algorithm performance are also discussed. In [77], an increasing sub-gradient algorithm of limited convex optimization is investigated and the convergence of the algorithm is certified mathematically.

In consensus strategy, each agent is negotiated to bring each expectation state to a common expected value by the network topology. Consensus originates from the field of biology. In the field of computer science, consensus is the theoretical basis for distributed computing and algorithmic implementation. For the wireless sensor network, a consensus-distributed estimation algorithm is designed in [71], which improves the estimation accuracy under the conditions of guarantee convergence. Based on the consensus protocol, a distributed estimation algorithm with connection noise of the ad hoc network is investigated in [66] and [67] respectively. By solving the convex optimization problem, the distributed estimation algorithm is not only improving estimation precision of network signal, but can also restrain the disturbance of noise effectively. A distributed $H_{\infty }$ filtering with consensus strategy is designed in [103], which can effectively suppress the tracking error of network signal through the algorithm iteration. Furthermore, the problem of distributed Kalman filtering is also investigated in [104].

Compared with the other two strategies, in IWSN, diffusion strategy has better convergence, better collected information from agent’s local neighborhoods, stronger robustness about the node and communication link, and it is easier to implement the distributed algorithm through the topology. According to a different construction of distributed estimation error, the diffusion strategy has a different form of algorithm implementation. Based on the mean-square error (MSE) algorithm, the minimum distributed least mean square (DLMS) estimation of the intelligent network is investigated in [105]. Furthermore, the diffusion DLMS algorithm is investigated and the optimality of the algorithm is also analyzed. On this basis, the diffusion LMS algorithm of time-varying parameters is designed in [106] and gives the proof of convergence and optimality. Furthermore, the implementation condition and the solution method of the diffusion LMS algorithm are studied in [107]. The stability and convergence of the algorithm are further analyzed in the paper.

Based on recursive least squares (RLS) algorithm, distributed RLS estimation algorithm for IWSN is investigated in [85], which guarantees global optimization of IWSN. For complex intelligent network systems, the RLS algorithm is more suitable for online estimation. Thus, an RLS algorithm with a local diffusion strategy is studied in [108]. The stability and convergence of the diffusion RLS algorithm is investigated in [109] and applies it to actual verification in ad hoc network with noisy in [86]. In the field of Kalman filter, a distributed Kalman filter with diffusion strategy is designed and the convergence and stability of the algorithm are also investigated in [98]. After comparing the diffusion strategy with the consensus strategy of two kinds of distributed estimation algorithms, reference [110] points out that diffusion strategy is more than the consensus strategy on convergence speed and stability performance.

The IWSN is an adaptive network system, which have the capability of sense, analysis, learning, judging, decision-making, and awareness [111]. In the process of online learning, intelligent network not only obtains the information about the environment in real-time, but also accumulates knowledge and makes decisions [112]. Based on adaptive diffusion strategy, the global optimization cost function is designed through all nodes on the network in [113]. According to agent interaction in the neighborhood, the algorithm successfully implements the IWSN distributed optimization and online learning.

Because there are a lot of online data in IWSN, in the process of distributed learning is easily getting into “curse of dimensionality”. To avoid this, a sparse distributed estimation algorithm is proposed based on diffusion LMS strategy in [83]. The validity of the algorithm is also verified by two different penalty functions. Based on the diffusion strategy, a combined project adapt protocol (CPAP) is designed in [114]. Using a robust statistics loss function, the CPAP, not only realizes the distributed estimation of the intelligent network, but also analyzes the robustness of intelligent node connection failure. In the implementation of distributed estimation, it requires analysis and processing a large amount of data in an intelligent network. In selecting data validity, a kind of data dimension reduction is designed to improve the execution efficiency of distributed algorithms in [115]. By the conjugate function and the dual decomposition, the problem of intelligent network learning is transformed into the problem of distributed optimization in [116]. Using the diffusion strategy, the online dictionary learning of IWSN is implemented.

The problems of distributed estimation theory are mainly include distributed algorithm designing, the convergence of the distributed algorithm, the computational complexity, and sparse algorithm of data, etc. [117]. On the basis of comparing the advantages and disadvantages of several kinds of distributed algorithms, the distribution optimization algorithm is reviewed and the prospect of further research direction of distributed optimization in [71]. Furthermore, a kind of distributed gradient algorithm is designed and the convergence rate about the algorithm is studied simultaneously in [118], which guarantee the distributed algorithm converges to the common expectation value on the basis of the total cost function of IWSN, which is equivalent to the sum of all the nodes’ cost functions.

For the problem of network utility maximization, the distributed Newton optimization algorithm of the intelligent network is designed using the matrix splitting method in [119] and in [120]. The realization process and convergence character of the distribution are also investigated in the two papers. Based on game theory, diffusion LMS algorithm with the ability of learning and self-optimization of intelligent network is investigated in [121]. At the same time, a framework of adaptive game learning theory is proposed and the distributed estimation convergence and stability are also analyzed. Based on graphical evolutionary game, under the regarding each intelligent point as each player, not only the data diffusion process is investigated, but also strategies of data evolution and data development are also analyzed in [122]. Furthermore, based on the diffusion strategy, the distributed Pareto optimization problem is also studied and multi-objective optimization of IWSN is realized in [123].

In addition, the actual IWSN is mixed with numerous networks among components. Under this case, the internal clock of each intelligent node is unable to complete synchronization. Therefore, each intelligent node cannot guarantee status updates at the same time. There are a lot of negative effects in asynchrony of an intelligent network, such as induced delay and switching topologies. Therefore, there is a need for further research asynchronous distributed estimation and optimization about the intelligent networks. The asynchronous learning model and stability condition of the intelligent network is proposed in [124]. On this basis, the performance about asynchronous mathematical model is also analyzed in [125]. Finally, after a comprehensive comparison of the performance of the synchronous and asynchronous algorithm, the advantages and disadvantages are pointed out respectively in [126]. Based on event-driven theory, a kind of distributed optimization strategy is analyzed in [127]. In the process of executing the distributed optimization algorithm, each agent can optimize the common goal’s function by cooperation strategy. When the network wireless communication energy is limited, the distributed algorithm not only ensures convergence of the optimization process, but also extends the communication life of IWSN [128].

## 5. Conclusions and Future Perspective

Combined characteristics of actual IWSN with development demanding of a distributed algorithm, this paper review the latest research achievement of distributed estimation and distributed learning in recent years. It is of very important practical significance that the nature of the distributed algorithm is intensively studied and understood. Furthermore, the main purpose of this paper is to further promote distributed estimation and dynamic optimization technology in the practical engineering application.

Although existing research shows that the distributed estimation of IWSN has been well developed with linear estimation, such as the LMS algorithm and the RLS algorithm, there are some problems of IWSN to investigate intensively, which include implementation process of online nonlinear estimation, the computational complexity and “curse of dimensionality” of distributed estimation algorithm, and the impact of the distributed estimation algorithm by the dynamic change of network topology. Therefore, these problems can be summarized the following three basic scientific questions about the intelligent network: (1) How to realize distributed adaptive mechanism through IWSN; (2) how to carry out a distributed estimation algorithm through IWSN; (3) how to achieve distributed optimization approach through IWSN.

In the future research, the distributed estimation and dynamic optimization should be focused on the following direction.

(1) Because the intelligent network system is a distributed self-organizing system, the online estimation and data updating are realized among agents by the network topology. How to realize distributed online estimation through network topology is one of the important research directions under the non-cooperation strategy.

(2) There are a lot of online data in an intelligent network, but how to manage these data with online sparse algorithm, to reduce the computational complexity, and to realize the distributed optimization are difficulties in the application of IWSN.

(3) Based on statistical learning theory and online kernel learning, it is very important to process the nonlinear and uncertainty of the network and to accomplish the online distributed kernel adaptive estimation algorithm of IWSN.

(4) Based on the theory of Markov differential game, the distributed robust optimization algorithm of IWSN is established, which not only can avoid solving the Nash equilibrium directly, but also can achieve the global optimal of the whole network system.

In view of the wide application of wireless sensors in IWSN, the limited network resources are restricted by many factors, which include power supply, data analysis, computational complexity, and communication bandwidth. Therefore, by integrated machine learning, distributed algorithm, control theory, deep reinforcement learning, parallel computation, online sparse algorithm, and dynamic optimization theory with engineering application, the distributed estimation and distributed learning have been new areas of scientific research of an intelligent network, which should be intensive research urgently in the big data environment.
